# Obituary

**Published:** 1893-12

**Authors:** 


### Obituary.

Dr. Charles Warrington Earle, of Chicago, died in the city of his home on Sunday, November 19, 1893, at the age of 48 years. He had been ill about four weeks, and, according to reports, his disease was spinal meningitis.

Dr. Earle was president of the Woman's Medical College ; one of the founders of the College of Physicians and Surgeons ; president of its board of directors ; professor of operative obstetrics in the Post-Graduate Medical School ; ex-president of the Illinois Medical Society, and a member of the British Medical Association.

Dr. Earle was well known by reason of his contributions to the literature of obstetrics and the diseases of children. After graduating in medicine in Chicago, he pursued his studies in Paris, Berlin, and Vienna. In 1871, he married Miss Fannie L. Bundy, a sister of the late Maj. J. M. Bundy, of the New York *Mail and Express*.

Dr. Earle's military career was almost phenomenal. He enlisted at the age of sixteen years, and before reaching his eighteenth birthday he was promoted to the rank of second lieutenant, and in this capacity commanded his company at the battle of Chicamauga, where, out of forty-five men of the company, it is reported that thirty-five were either killed or wounded. Official reports of the battle made special mention of his bravery. Two days afterward the army retreated into Chattanooga. By some mischance, Lieut. Earle and thirteen others were left on Mission Ridge, where they were captured and taken to Richmond. He escaped from Libby prison through the famous tunnel, and after a week of wandering in the Virginia woods, reached the Union lines near Williamsburg. Returning to his own command, he was promoted to first lieutenant, and commanded his company much of the time through the Atlanta campaign. Dr. Earle was a splendid specimen of robust manhood, and has sadly and suddenly been stricken down in the very prime of his usefulness.

---

Sir Andrew Clark, Bart., M. D., F. R. S., LL. D., died in London, November 6th, in the 68th year of his age. He was one of the best known medical men of England, and his writings were numerous and valuable.

---

Dr. John M. Keating, LL. D., formerly of Philadelphia, died at Colorado Springs, his late residence, on Saturday, November 18, 1893. Dr. Keating was formerly medical director of the Pennsylvania Mutual Life Insurance Company, of Philadelphia, was an author of national reputation, and a physician of distinction. One of his latest literary efforts was the editing of a series of International Clinics, issued from the press of the J. B. Lippincott Co., of Philadelphia.

